# Reporting quality of randomized controlled trials in prehabilitation: a scoping review

**DOI:** 10.1186/s13741-023-00338-8

**Published:** 2023-08-31

**Authors:** Dominique Engel, Giuseppe Dario Testa, Daniel I. McIsaac, Francesco Carli, Daniel Santa Mina, Gabriele Baldini, Celena Scheede-Bergdahl, Stéphanie Chevalier, Linda Edgar, Christian M. Beilstein, Markus Huber, Julio F. Fiore, Chelsia Gillis

**Affiliations:** 1 Department of Anaesthesiology and Pain Medicine, Inselspital, Bern University Hospital, University of Bern, Bern, Switzerland; 2 Department of Anesthesia, McGill University, Montréal, QC Canada; 3 Division of Geriatric and Intensive Care Medicine, University of Florence and Azienda Ospedaliero Universitaria Careggi, Florence, Italy; 4 Clinical Epidemiology Program, Department of Anesthesiology and Pain Medicine, Ottawa Hospital Research Institute, School of Epidemiology and Public Health, University of Ottawa, Ottawa, ON Canada; 5 Department of Anesthesia and Pain Management, Faculty of Medicine, Faculty of Kinesiology and Physical Education, University Health Network, University of Toronto, Toronto, Ontario Canada; 6 Section of Anesthesiology, Intensive Care and Pain Medicine, Anesthesiology and Intensive Care Department of Health Sciences, University of Florence, Florence, Italy; 7 Department of Kinesiology and Physical Education, McGill University, Montreal, QC Canada; 8 School of Human Nutrition, McGill University, Sainte-Anne-de-Bellevue, Quebec, H9X 3V9 Canada; 9 Department of Medicine, Research Institute of the McGill University Health Centre, McGill University, Montreal, QC Canada; 10 Prehabilitation Clinic, Montreal General Hospital, McGill University Health Centre, Montreal, Quebec Canada; 11 Department of Surgery, McGill University, Montreal, QC H3G 1A4 Canada

**Keywords:** Prehab, Pre-rehab, Perioperative medicine, ERAS (enhanced recovery after surgery)

## Abstract

**Background:**

Inadequate study reporting precludes interpretation of findings, pooling of results in meta-analyses, and delays knowledge translation. While prehabilitation interventions aim to enhance candidacy for surgery, to our knowledge, a review of the quality of reporting in prehabilitation has yet to be conducted. Our objective was to determine the extent to which randomized controlled trials (RCTs) of prehabilitation are reported according to methodological and intervention reporting checklists.

**Methods:**

Eligibility criteria: RCTs of unimodal or multimodal prehabilitation interventions. Sources of evidence: search was conducted in March 2022 using MEDLINE, Embase, PsychINFO, Web of Science, CINAHL, and Cochrane. Charting methods: identified studies were compared to CONSORT, CERT & Modified CERT, TIDieR, PRESENT, and CONSORT-SPI. An agreement ratio (AR) was defined to evaluate if applicable guideline items were correctly reported. Data were analyzed as frequency (*n*, %) and mean with standard deviation (SD).

**Results:**

We identified 935 unique articles and included 70 trials published from 1994 to 2022. Most prehabilitation programs comprised exercise-only interventions (*n* = 40, 57%) and were applied before oncologic surgery (*n* = 32, 46%). The overall mean AR was 57% (*SD*: 20.9%). The specific mean ARs were as follows: CONSORT: 71% (*SD*: 16.3%); TIDieR: 62% (*SD*:17.7%); CERT: 54% (*SD*: 16.6%); Modified-CERT: 40% (*SD*:17.8%); PRESENT: 78% (*SD*: 8.9); and CONSORT-SPI: 47% (*SD*: 22.1).

**Conclusion:**

Altogether, existing prehabilitation trials report approximately half of the checklist items recommended by methodological and intervention reporting guidelines. Reporting practices may improve with the development of a reporting checklist specific to prehabilitation interventions.

**Supplementary Information:**

The online version contains supplementary material available at 10.1186/s13741-023-00338-8.

## Background

Prehabilitation is the approach of enhancing the functional capacity of individuals to enable them to withstand a stressful event (Mayo et al. 2011). Prehabilitation programs vary but are generally designed to prepare patients for the impending physiological stress of surgery, through a combination of exercise, nutrition, and medical management (e.g., smoking cessation), so that these stronger patients experience an improved recovery. While the concept is intuitive, practice and evidence have been variable. A recent umbrella review of 55 systematic reviews, including 1412 unique articles, identified that surgical prehabilitation likely improves both functional and clinical outcomes, but the certainty of the evidence was mostly low (McIsaac 2022). These inconsistent findings could be, in part, related to the heterogeneity of study populations, designs, interventions, and outcomes that often cannot be melded together into one clear message regarding prehabilitation (Gillis et al. 2021). In addition, poor quality of reporting in previous trials may have hampered appropriate study quality assessment and interpretation of findings (Candy et al. 2018).

A scoping review of 37 nutrition-related prehabilitation studies in oncology identified that reporting of the nutrition component was inadequate and inconsistent. For instance, one-quarter of the studies included a nutrition intervention that was indiscernible, and two-thirds did not monitor program adherence (Gillis et al. 2021). These are just two examples of a common failure to clearly and thoroughly report healthcare research (Scales et al. 2008; Lai et al. 2007; Chan and Altman 2005). Reporting guidelines, such as the Consolidated Standards of Reporting Trials (CONSORT) (Moher et al. 2010), were developed to ensure that research studies are reported in a complete, transparent, and accurate manner. Inadequate research reporting is problematic for several reasons:

1. If authors do not provide sufficient study details, readers are left with an incomplete picture of the research methods and interventions tested. As such, it is not possible to critically appraise the work, judge the trustworthiness of the results, and draw appropriate conclusions.
2. Poor reporting hinders adequate meta-analyses of results from different studies. This can limit the overall evidence base to inform clinical practice and policy.
3. Without adequate reporting, study findings cannot be accurately replicated in practice nor in future research. This can reduce the overall reliability of the evidence base.
4. Poor reporting can lead to inconsistencies or errors in the interpretation of study results. This can reduce confidence in the findings and make it harder for policymakers, clinicians, and patients to make evidence-based decisions. For the reasons stated above, there are ethical and moral reasons for reporting research adequately.

Improving quality of prehabilitation research and the certainty of evidence for prehabilitation requires the conduct and reporting of methodologically robust clinical trials to expected standards (Yamato et al. 2016; Merkow et al. 2021). To date, no study has systematically appraised the extent to which prehabilitation trials are reported according to available guidelines. This is an important step to help us understand how current reporting practices are contributing to the evidence base of prehabilitation and to identify gaps in reporting that could be addressed to improve the quality of evidence in this field. Therefore, we conducted a scoping review to evaluate the quality of reporting of randomized trials focused on prehabilitation.

## Methods

Scoping reviews are carried out to identify the types of available evidence in a given field, clarify key concepts and definitions in the literature, examine how research is conducted on a certain topic or field, identify key characteristics or factors related to a concept, and sometimes as a precursor to a systematic review (Munn et al. 2018). In contrast to systematic reviews, scoping reviews do not aim to critically appraise or synthesize results to a particular question but rather aim to provide an overview or map of the available evidence.

We performed a scoping review of the literature based on the framework outlined by Arksey and O’Malley (Arksey and O'Malley 2005), recommendations of Levac et al. (Levac et al. 2010), and in accordance with the Preferred Reporting Items for Systematic reviews and Meta-Analysis extension for Scoping Reviews (PRISMA-ScR). The review included the following five key phases: (1) identifying the research question, (2) identifying relevant studies, (3) selecting studies, (4) charting the data, and (5) collating, summarizing, and reporting the results (Arksey and O'Malley 2005). A project team consisting of health researchers and health providers was established to inform the review design, conduct, and interpretation.

### Identifying the research question

The main objective of this scoping review was to provide an overview of the quality of reporting in primary studies providing the highest level of evidence (Howick et al. 2011 ), randomized controlled trials (RCTs), in prehabilitation and to generate recommendations based on identified gaps. Our specific research question was as follows: To what extent do prehabilitation RCTs adhere to reporting guidelines focused on the following: (1) RCT methods (Consolidated Standards of Reporting of Trials, CONSORT 2010) (Schulz et al. 2010), (2) interventions (TIDieR, template for intervention description and replication 2014) (Hoffmann et al. 2014), (3) therapeutic exercise interventions (Consensus on Exercise Reporting Template, CERT 2016 (Slade et al. 2016), and Modified-CERT 2017 (Page et al. 2017)), (4) exercise and nutritional interventions (Proper Reporting of Evidence in Sport and Exercise Nutrition Trials (Betts et al. 2020), PRESENT 2020) and (5) psychosocial interventions (CONSORT 2010 extension for psychosocial interventions (Montgomery et al. 2018), CONSRT-SPI 2018). A brief description of each targeted guideline can be found in Table 1, and a list of guideline items can be found in Supplementary Material 1.

**Table 1:** Brief description of existing reporting guidelines used in our scoping review

**CONSORT**	The CONSORT statement includes a 25-item checklist. It provides guidance for reporting all randomized controlled trials but focuses on the most common design type — individually randomized, two groups, parallel trials (Schulz et al. 2010)
**TIDieR**	The purpose of the 12-item TIDieR checklist is to prompt authors to describe interventions in sufficient detail to allow their replication. The checklist contains the minimum recommended items for describing interventions (Hoffmann et al. 2014)
**CERT**	The CERT, a 16-item checklist, is designed to improve the reporting of exercise programs in all evaluative study designs and contains 7 categories: materials, provider, delivery, location, dosage, tailoring, and compliance (Slade et al. 2016)
**Modified-CERT**	While the CERT is specific to exercise interventions, therapeutic exercise may need even more detail for clinical implementation or replication. The supplement provides further guidance on reporting therapeutic exercise intervention within the context of the CERT checklist (Page et al. 2017)
**PRESENT**	The PRESENT, a 34-item checklist, has been adapted from the CONSORT guidelines to specifically address the unique combination of challenges and opportunities facing researchers within the broad fields of sports nutrition and exercise metabolism (Betts et al. 2020)
**CONSORT-SPI**	The CONSORT-SPI checklist extends 9 of the 25 items from CONSORT 2010: background and objectives, trial design, participants, interventions, statistical methods, participant flow, baseline data, outcomes and estimation, and funding. Additionally, an item related to stakeholder involvement and the flow diagram related to participant recruitment and retention were edited (Montgomery et al. 2018)

Consolidated Standards of Reporting of Trials, *CONSORT 2010*; *TIDieR*, Template for intervention description and replication 2014; Consensus on Exercise Reporting Template, *CERT 2016*; Modified-CERT 2017; Proper Reporting of Evidence in Sport and Exercise Nutrition Trials, *PRESENT 2020*; CONSORT Extension for Psychosocial Interventions, *CONSRT-SPI 2018*

### Identifying relevant studies

Given that our goal was to map the quality of reporting of prehabilitation RCTs, we first focused our scoping review on published “prehabilitation”-labelled RCTs in which the prehabilitation intervention was randomly assigned, independent of the type and method of randomization. We then included studies that met the following working definition of prehabilitation as described in previous literature (Scheede-Bergdahl et al. 2019; Gillis et al. 2018; Luther et al. 2018; McIsaac 2022):

A unimodal intervention consisting of exercise, nutrition or cognitive/psychological training, or a multimodal intervention that combines exercise, nutrition and/or cognitive/psychological training with or without other interventions, undertaken for seven or more days before surgery (which is a period consistent with Enhanced Recovery After Surgery initiatives, not prehabilitation) to optimize a patient's preoperative condition and improve postoperative outcomes.

A search strategy was developed with the assistance of a librarian (G. G.; Supplementary Material 2) in accordance with the Peer Review of Electronic Search Strategy process (McGowan et al. 2016). We used broad search terms that encompassed the following: prehab* or pre-hab* or prerehab* or pre-rehab* or (preoperative* or pre-operative*) adj rehab*) AND randomized controlled trial. All studies after 1946 were included (no date restriction). The final search was conducted on March 25, 2022, using MEDLINE, Embase, PsychINFO, Web of Science, CINAHL, and Cochrane, and was limited to French and English. Hand searching the reference lists of key papers, including all identified systematic reviews and meta-analyses of prehabilitation, was also conducted.

### Study selection

Two reviewers (D. E. and G. T.) independently reviewed titles and abstracts for inclusion by using the Rayyan web application (www.rayyan.ai, Cambridge, MA 02142, USA). Articles were considered for full-text review if inclusion criteria were met: (1) trials delivering a surgical “prehabilitation”-labelled program for adult patients (aged > 18 years) and in accordance with the above definition and (2) were RCTs (including pilot RCTs). Studies were excluded if they were narrative reviews or editorials, systematic reviews, meta-analyses, scoping reviews, pooled analyses, secondary analyses, study protocols, consensus guidelines, conference abstracts, publications not in English or French, or involved pre-surgical treatment not related to prehabilitation. As an example, isolated preoperative risk factor management (e.g., smoking cessation, anemia treatment, medication management in isolation) and interventions applied immediately (i.e., < 7 days) before surgery were excluded. The two reviewers (D. E. and D. T.) then independently reviewed selected articles for full-text review. Disagreements were addressed by discussion and consensus.

### Charting the data

Interventions were charted as exercise if they consisted of either endurance/aerobic exercise to increase functional capacity, strengthening/resistance to increase muscle mass, flexibility, or balance exercises as well as a combination thereof. An intervention was considered a nutrition intervention, when it was stated as such and aimed to improve nutritional state or dietary intake. Meditation and breathing exercises to achieve mindfulness or reduce emotional stress were considered as psychosocial interventions, whereas inspiratory muscle training (IMT) was considered functional training (i.e., increasing the functionality/efficiency of breathing and coughing). A program was considered multimodal when two or more modalities were performed concurrently.

The checklist items of CONSORT 2010 (37 items: item 2b was split into objectives and hypotheses), CERT (16 items), Modified-CERT (16 items), TIDieR (12 items), PRESENT 2020 (34 items), and CONSORT-SPI 2018 (14 items) were then used to evaluate the reporting of methods and interventions accordingly. All studies were compared to CONSORT 2010 and TIDieR. Exercise interventions were compared to CERT and Modified-CERT. If a program comprised exercise and nutrition, the PRESENT 2020 guideline was applied. Psychosocial interventions were compared to CONSORT-SPI 2018. CONSORT 2010 item 3b (important changes to methods after trial commencement), 6 (changes to trial outcomes after the trial commenced, with reasons), and 7b (explanation of any interim analyses and stopping guidelines) are reported as described because of the inability to determine whether these items were not applicable or not reported. Two researchers (D. E. and G. T.) independently extracted and compared data for the first five studies to ensure consistent data extraction before completing the remaining extraction autonomously. The extraction process included the main manuscript as well as all referenced protocols and available supplementary material. Ultimately, after finalization, disagreements were clarified with the senior author (C. G.).

A data extraction template (Excel, Microsoft 2010, Redmond, WA, USA) was developed in consultation with the project team and included study (e.g., year, origin, sample size, and primary outcome classified according to Walton et al. (Walton et al. 2015)), population (e.g., type of surgery, cancer type), and intervention (e.g., type of program, duration) characteristics. Prevalence of reporting of malnutrition, frailty, and sarcopenia was also documented.

### Collating and summarizing results

Similar to methods used in previous studies examining quality of reporting (McCambridge et al. 2021; Yamato et al. 2018), we assessed completeness of study reporting by creating *a sum score* for every checklist item that was equal to the number of studies the guideline was compared to (e.g., every study was compared to CONSORT). The *applicability index (AI)* for every checklist item was then calculated as follows: if an item was considered “not applicable,” that point was subtracted from the *sum score* for that particular checklist item to obtain the AI. The agreement ratio (AR) — based on the AI — was defined as how many times a guideline item was correctly reported, with 100% indicating every study reported this item adequately. For example, consider the completeness of reporting for item 9 of the CERT checklist (i.e., content of any home-based program). If 50 studies included an exercise intervention and thus could be compared to the CERT checklist (yields *sum score*), but 25 of these studies did not include a home-based component, this item was not deemed underreported, it was deemed “not applicable”; as a result, 25 should be subtracted from the sum score to create an AI of 25. If 20 of the remaining studies scored “yes” for reporting this item correctly, this would yield an AR of 20/25 = 80% for item number 9 of the CERT checklist. Calculations were done with Excel (Microsoft, Redmond, USA). Mean agreement was evaluated overall, and by decade (data permitting): 1993–2003, 2004–2013, and 2014–2022, to evaluate evolution in reporting quality.

Data were analyzed using descriptive statistics: frequencies (*n*, %), range (min–max), mean and standard deviation (SD) for normally distributed data, or median and interquartile range [IQR] if the data were not normally distributed. These computations were performed with R version 4.0.2 (the R Core Team [2020], R: a language and environment for statistical computing. R Foundation for Statistical Computing, Vienna, Austria). All calculations were verified by a statistician (M. H.) to ensure that scoring was accurate. The study team was consulted to provide input regarding the interpretation of the findings, identification of research gaps, and venues for future research.

## Results

### Search results

**Fig. 1:**
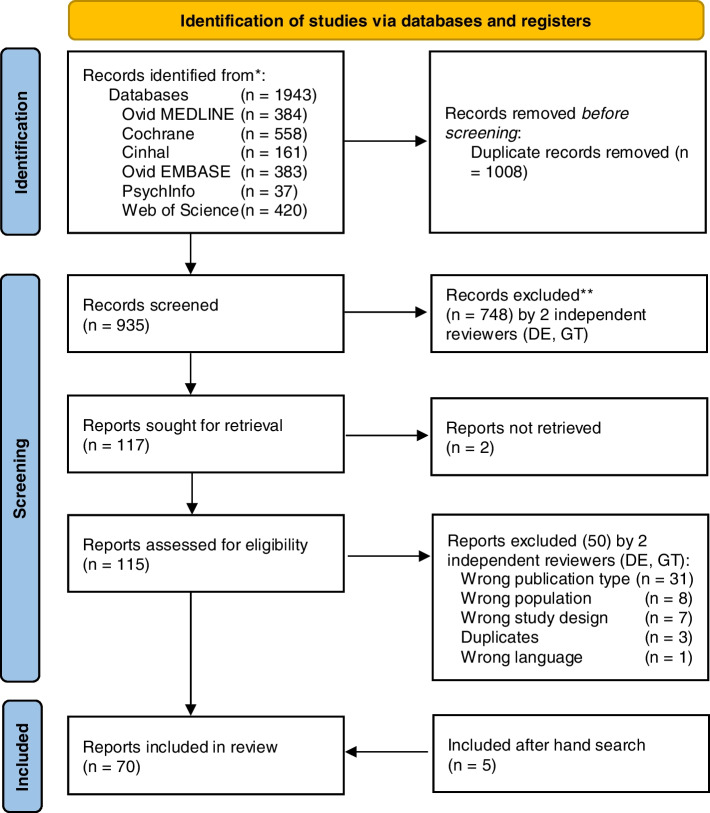
PRISMA flow diagram

Our search identified 935 unique articles (PRISMA diagram presented in Fig. 1). After abstract screening, 117 articles were suitable for full-text review, 2 of which were not accessible, and 50 articles were excluded because of publication type (*n* = 31), population (*n* = 8), study design (*n* = 7), duplicates (*n* = 3), and language (*n* = 1), leaving 65 articles. Hand searching produced 5 additional articles, resulting in 70 articles included in this review (An, et al. 2021; Argunova et al. 2021; Ausania et al. 2019; Barberan-Garcia, et al. 2018; Berkel, et al. 2022; Blackwell et al. 2020; Bousquet-Dion et al. 2018; Brown et al. 2014; Calatayud et al. 2017; Carli, et al. 2020; Carli et al. 2010; D'Lima et al. 1996; Dunne et al. 2016; Ferreira et al. 2021; Ferreira et al. 2021; Fulop et al. 2021; Gillis et al. 2014; Gillis et al. 2016; Gloor, et al. 2022; Granicher et al. 2020; Grant et al. 2017; Gravier et al. 2022; Huang et al. 2017; Huang et al. 2012; Hulzebos et al. 2006; Humeidan et al. 2021; Jahic et al. 2018; Jensen et al. 2015; Kim et al. 2009; Kim, et al. 2021; Lai et al. 2017; Lai et al. 2017; Liang et al. 2018; Licker et al. 2017; Lindback et al. 2018; Liu et al. 2020; Lopez-Rodriguez-Arias et al. 2021; Lotzke et al. 2019; March et al. 2021; Mat Eil Ismail et al. 2016; Matassi et al. 2014; McKay et al. 2012; Minnella et al. 2021; Minnella et al. 2018; Minnella et al. 2020; Morano et al. 2013; Nguyen et al. 2022; Nielsen et al. 2010; Northgraves et al. 2020; O'Gara et al. 2020; Onerup et al. 2022; Peng et al. 2021; Rooks et al. 2006; Santa Mina et al. 2018; Satoto et al. 2021; Sawatzky et al. 2014; Sebio Garcia et al. 2017; Shaarani et al. 2013; Steinmetz et al. 2020; Tenconi et al. 2021; Topp et al. 2009; Vagvolgyi et al. 2018; VE et al. 2021; Waller et al. 2020; Woodfield, et al. 2022; Yamana et al. 2015; Beaupre et al. 2004; McIsaac et al. 2022; Brown et al. 2012; Cavill et al. 2016).

### Prehabilitation study characteristics

Table 2 describes the intervention and patient characteristics for all included studies. The period of publication ranged from 1995 to 2022 with an increase in recent years. Of the 70 studies, 46% of the trials were conducted in Europe (*n* = 32), 36% in North America (*n* = 25), and 16% in Asia (*n* = 11). The number of participants ranged from 15 to 668 (mean (SD): 89.8 (93.2); median [IQR]: 60.0 [34.0–110.0]). We noted that a sample size calculation was not reported in 21% of trials (*n* = 15). Of those trials with a sample size calculation, 44% reached their target (*n* = 24), and a positive finding was attained for the primary outcome in 64% (*n* = 45) of trials. The primary outcome was 36% performance based (*n* = 25), 31% clinician reported (*n* = 22), 14 % patient reported (*n* = 10), 4% observer reported (*n* = 3), and 13% mixed or unspecified (*n* = 9). Duration of the prehabilitation program ranged from 1 to 14 weeks (mean (SD): 4.7 (2.5), median [IQR]: 4.0 [3.0–6.0]), with 3 to 126 exercise sessions (mean (SD): 18.8 (17.3); median [IQR]: 14.0 [11.2–20.8]). Offered programs were exercise only in 57% (*n* = 40) of trials, 33% (*n* = 23) of trials were multimodal, nutrition/function-only each accounted for 3% (*n* = 2), and psychosocial comprised 4% (*n* = 3) of published trials. Prehabilitation was applied in 46% of trials for oncologic surgery (colorectal, lung, and urological; *n* = 32), 43% for general surgery patients (orthopedic, heart, and lung; *n* = 30%), and in 11% of trials as a mixed cohort (oncologic and non-oncologic). Screening for malnutrition was reported in 11% (*n* = 8), frailty in 6% (*n* = 4), and the incidence of sarcopenia not once. Finally, 19 trials cited the CONSORT 2010 guideline, 1 trial cited TiDieR, and 1 cited CERT.

**Table 2:** Study, surgery, and intervention characteristics of 70 randomized trials of prehabilitation

**Study characteristics**		
**Year of publication, *n* (%)**	70	100%
1995	1	1%
2004	1	1%
2006	2	3%
2009	2	3%
2010	2	3%
2011	1	1%
2012	2	3%
2013	3	4%
2014	6	9%
2015	2	3%
2016	4	6%
2017	5	7%
2018	7	10%
2019	3	4%
2020	9	13%
2021	13	19%
2022	7	10%
**Origin of studies, *n* (%)**	70	100%
Europe	32	46%
North America	25	36%
Asia	11	16%
Australia	1	1%
South America	1	1%
**Type of prehabilitation program, *n* (%)**	70	100%
Exercise only	40	57%
Nutrition only	2	3%
Functional	2	3%
Cognitive training	3	4%
Multimodal	23	33%
**Population included, *n* (%)**	70	100%
Surgery, non-oncologic	30	43%
Surgery, oncologic	32	46%
Mixed oncologic and non-oncologic	8	11%
**Surgery characteristics**^1^**, *n* (%)**		
*Non-oncological surgery*	38	100%
Orthopedic	19	50%
Heart	6	16%
Spine	4	11%
Colorectal	3	8%
Lung	1	3%
Hernia	1	3%
Mixed non-oncologic surgeries	4	11%
*Oncologic surgery*	40	100%
Colorectal	14	35%
Lung	12	30%
Urological	4	10%
Esophageal	2	5%
Hepatobiliary	1	3%
Pancreatic	1	3%
Mixed oncologic surgeries	6	15%
**Sample size, *n* (%)**	70	100%
Reached	24	34%
Not reached	31	44%
Not calculated	15	21%
**Number of patients per trial**		
Min–max number	15–668	
Mean (SD)	89.8 (93.2)	
Median [IQR]	60.0 [34.0–110.0]	
**Primary outcome, *n* (%)**	70	100%
Performance based	25	36%
Clinician reported	22	31%
Patient reported	10	14%
Observer reported	3	4%
Biomarker	1	1%
Mixed	5	7%
Unclear/not specified	4	6%
**Primary outcome significant, *n* (%)**	70	100%
Yes	45	64%
No	25	36%
**Baseline reporting of patient characteristics, *n* (%)**	70	100%
Malnutrition	8	11%
Frailty	4	6%
Sarcopenia	0	0%
**Intervention characteristics**		
**Location of prehabilitation delivery, *n* (%)**	70	100%
Home	20	29%
Supervised	28	40%
Tele-prehab	1	1%
Combination	19	27%
Not specified	2	3%
**Nutrition intervention, *n* (%)**	70	100%
Yes	16	23%
No	51	73%
Usual care nutrition	3	4%
**Psychological intervention, *n* (%)**	70	100%
Yes	13	19%
No	57	81%
**Duration of prehabilitation (weeks)**		
Min–max	1–14	
Mean (SD)	4.7 (2.5)	
Median [IQR]	4.0 [3.0–6.0]	
**Total prehabilitation sessions**		
Min–max	3–126	
Mean (SD)	18.8 (17.3)	
Median [IQR]	14.0 [11.2–20.8]	
**Guidelines cited**	*N*	100%
CONSORT	17	24%
CONSORT & TIDieR	1	1%
CONSORT & CERT	1	1%
CONSORT flow chart	12	17%

^1^Because 8 studies included both cancer and non-cancer patients, surgery characteristics overlap. *Min* Minimal value; *Max* Maxima value, *SD* Standard deviation, *IQR* Interquartile range, *ns* Not specified, *CONSORT* Consolidated Standards of Reporting of Trials, *TIDieR* Template for Intervention Description and Replication, *CERT* Consensus on Exercise Reporting Template

### Agreement with existing guidelines

**Fig. 2:**
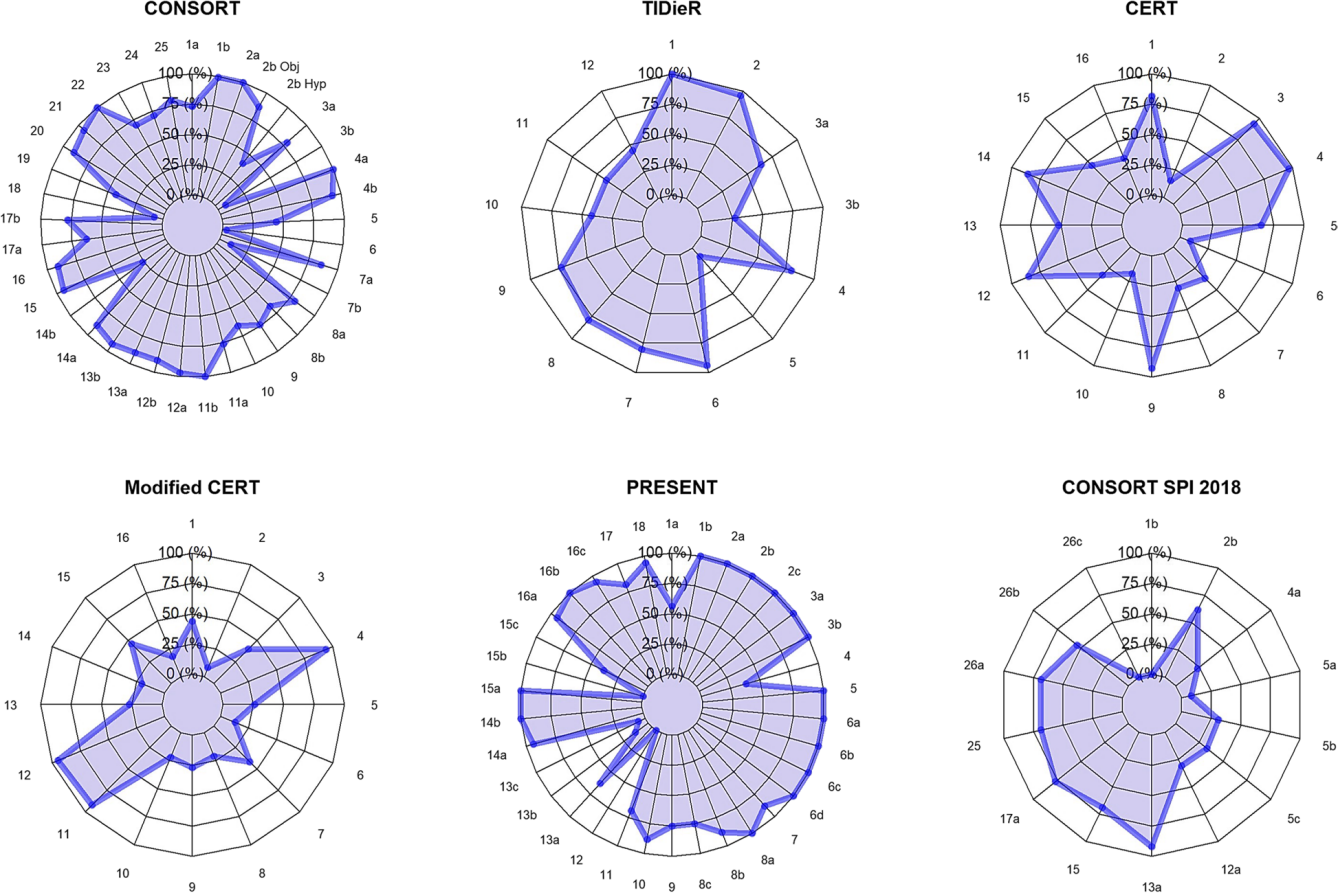
Spider graphs of the agreement ratios between CONSORT 2010, TIDieR 2014, CERT 2016, Modified-CERT 2017, PRESENT 2020, CONSORT-SPI 2018, and 70 randomized controlled trials of prehabilitation. Checklist items can be found in Supplementary material 1

We calculated sum scores for each item of every checklist: CONSORT 2010 and TIDieR were applied to 70 trials; CERT and Modified CERT, 65 trials; PRESENT 2020, 16 trials; and CONSORT-SPI 2018, 13 trials. The mean (SD) agreement ratio for all studies to all guideline items was 57% (20.9) with a range of 40 to 78%. Agreement by decade can be found in Supplementary material 3. Agreement ratios for all trials to the applicable guidelines are represented in Fig. 2 and Table 3.

**Table 3:** Agreement ratio for 70 randomized controlled trials of prehabilitation and 6 reporting guidelines

Author	Year	Program	CONSORT	CERT	Modified-CERT	TIDieR	PRESENT	CONSORT-SPI
An J. et al.	2021	0	74	56	47	54		
Argunova et al.	2022	0	31	46	33	46		
Ausania et al.	2019	4	40	19	13	23	56	
Barberan-Garcia at al.	2018	0	83	80	71	69		
Berckel et al.	2022	0	78	63	50	62		
Blackwell et al	2019	0	83	77	64	92		
Bousquet-Dion et al.	2018	4	75	50	40	69	76	77
Brown et al.	2013	0	65	56	47	77		
Brown et al.	2012	0	46	44	38	77		
Calatayud et al.	2016	0	71	44	43	46		
Carli et al.	2020	4	78	50	40	77	85	43
Carli et al.	2010	0	67	75	47	77		
Dunne et al.	2014	0	81	40	43	46		
Ferreira et al.	2020	4	89	88	80	92	85	69
Cavill et al.	2015	0	81	50	31	46		
Ferreira et al.	2021	4	70	63	27	77	76	43
Fulop et al.	2021	4	72	36	29	42	82	7
Gillis et al.	2014	4	84	56	67	69	79	21
Gillis et al.	2014	1	89			77	85	
Gloor et al.	2022	0	76	31	20	46		
Gränicher et al.	2020	4	77	73	57	85		
Grant et al.	2017	0	80	60	69	69		
Gavier et al.	2021	0	68	53	40	54		
Huang J. et al.	2017	4	75	53	36	62		14
Huang S. W. et al.	2011	0	58	25	20	31		
Humeidan et al.	2021	5	89			62		
Jahic et al.	2018	0	19	31	7	31		
Jensen et al.	2014	0	78	44	20	69		
Kim et al.	2009	0	49	50	20	69		
Kim et al.	2021	0	63	27	25	46		
Lai et al.	2016	4	66	33	27	46		
Linang et al.	2018	4	83	50	27	46	79	
Licker et al.	2016	0	92	53	33	54		
Lindbäck et al.	2017	0	77	47	57	69		
Liu et al.	2020	4	83	75	73	85	82	62
Lopez et al.	2021	4	56	31	13	46	62	62
Lotzke et al.	2019	5	84			54		
Marchand et al.	2021	0	94	73	71	100		
Mat Eli Ismail et al.	2016	0	56	40	27	50		
Matassi et al.	2014	0	68	64	47	73		
McIsaac et al.	2022	0	86	63	50	77		
McKay et al.	2012	0	69	67	50	69		
Minnella et al.	2021	4	78	73	33	85	85	64
Minnella et al.	2018	4	86	73	40	69	82	
Minnella et al.	2020	4	89	64	47	77	85	64
Morano et al.	2013	4	58	25	27	46		
Nguyen et al.	2022	0	86	63	50	75		
Nielsen et al.	2010	0	78	38	27	54		
Northgraves et al.	2020	0	77	87	73	92		
O'Gara et al.	2020	5	78			62		
Onerup et al.	2022	4	86	75	47	46		
Peng et al.	2021	0	69	67	55	73		
Sana Mina et al.	2018	0	74	56	27	62		
Tenconi et al.	2021	4	77	38	19	50		
Satoto et al.	2021	3	43	50	20	50		
Sawatzky et al.	2014	0	66	60	19	69		
Garcia et al.	2017	4	83	73	50	69		
Shaarani et al.	2013	0	49	63	60	77		
Steinmetz et al.	2020	0	64	53	50	46		
Topp et al.	2009	0	34	44	27	42		
Vagvolgyi et al.	2018	0	70	47	20	31		
Hemink et al.	2020	1	78			67	68	
Waller et al.	2022	4	78	56	20	54	76	36
Lai et al.	2017	4	74	47	29	38		
Woodfield et al.	2021	0	83	80	69	92		
Yamana et al.	2015	4	64	40	31	31		
D'Lima et al.	1995	0	22	27	29	31		
Rooks et al.	2006	0	56	53	33	77		
Beaupre et al.	2004	0	60	53	50	62		
Hulzebos et al.	2006	3	86	81	67	77		

Type of program: exercise, 0; nutrition, 1; psychosocial, 2; functional, 3; multimodal, 4; cognitive training, 5. Consolidated Standards of Reporting of Trials, *CONSORT 2010*; Template for Intervention Description and Replication, *TIDieR 2014*; Consensus on Exercise Reporting Template, *CERT 2016*; Modified-CERT 2017; Proper Reporting of Evidence in Sport and Exercise Nutrition Trials, *PRESENT 2020*; CONSORT Extension for Psychosocial Interventions, *CONSRT-SPI 2018*

### CONSORT 2010

The overall mean (SD) agreement with CONSORT 2010 guideline was 71% (16.3) and ranged between 19 and 94%. Mean agreement increased over time: 1993–2003: 22% (−) [*n* = 1], 2004–2013: 60% (13.9) [*n* = 13], and 74% (14.4) [*n* = 56]. Specific objectives or hypotheses (item 2b) were formulated in 64% of studies (hypotheses alone in 41%). Items regarding randomization (8a), randomization type (8b), allocation concealment (9) and its implementation, (10) and details about blinding (11a) had 65–80% agreement. Items reported with an agreement of 100% were background and explanation of the rationale (2a), eligibility criteria for participants (4a), interpretation of findings (22), and description of differences between interventions (11b).

### TIDieR 2014

Of the 70 prehabilitation trials, mean (SD) agreement with TIDieR was 62% (17.7) and ranged from 23 to 100%. Mean agreement varied little over time: 1993–2003: 31% (−) [*n* = 1], 2004–2013: 64% (16.0) [*n* = 13], 2014–2022: 62% (17.9) [*n* = 56]. Background and specific training of the provider (5) were the least reported, with 8% of trials reporting this item. Where materials used in the intervention can be accessed (3b) was mentioned in 27% of trials and description of such materials in 64% of trials. Mode of delivery (6) and rationale or goal of the intervention (2) were reported in 94% and 96% of trials, respectively. The brief name of the intervention (1) was reported in 100% of trials.

### CERT 2016

Sixty-five trials included an exercise intervention and were compared to the CERT guidelines. Mean (SD) agreement was 54% (16.6) and ranged from 19 to 88%. Mean agreement varied little over time: 1993–2003: 26.7 (−) [*n* = 1], 2004–2013: 51% (17.2) [*n* = 13], and 2014–2022: 55% (16.2) [*n* = 51]. The following items were least reported: details of motivation strategies (9%) (6), qualifications or specific training or experience of the instructor (15%) (2), and explanations for the non-exercise components of an intervention (18%) (10). Occurrence or management of adverse events (11) was reported in 33% of trials. Most reported items (> 90% of trials) were content of any home program (9), if exercises were offered in groups or for individuals (3), and if the interventions were supervised or not (4).

### Modified-CERT 2017

The 65 trials compared to the modified-CERT guideline attained a mean (SD) agreement of 40% (17.8) with a range of 7–80%. Mean agreement varied little over time: 1993–2003: 29% (−) [*n* = 1], 2004–2013: 39% (15.2) [*n* = 13], and 2014–2022: 41% (18.7) [*n* = 51]. Details on how each therapist was trained (2) was only reported in 8% of trials. Behavioral strategies (6), defined markers of success (16), and how exercises were tailored (14) were reported in 10–20% of all studies. Mode of delivery (4), limitations and future research considerations (11), and which exercises were in clinic and/or home (12) were reported in more than 50% of the studies.

### PRESENT 2020

Of the 16 studies with a combined exercise and nutrition intervention, the overall mean (SD) compliance to PRESENT 2020 was 78% (8.9) and ranged from 56 to 85%. The items order effects (12) and individual data (15b) of patients were never reported. Adjustments for violated statistical assumptions (13c) were reported in 6% of trials and additional unplanned analyses (13b) in 13% of trials. Only 38% of trials reported relevant harms (15c) and stated ethical approval or citing the Declaration of Helsinki (4).

### CONSORT-SPI 2018

Of the 13 studies with a psychological component, mean (SD) agreement to the CONSORT 2010-SPI 2018 guideline was 47% (22.1) with a range of 7–77%. Item 1b (reference to appropriate CONSORT 2010 extension) and 26c (incentives offered) were never reported. Item 5a, referring to the extent to which interventions were delivered by providers and taken up by participants as planned, was reported in 8% of trials. Items 4a (provider/setting), 5b (where information material about the intervention can be accessed), 12a (how missing data was handled), and 5c (how the providers were assigned) were reported in 23–33% of trials. The most frequently reported item was 13a (participant flow) with 92% of trials including this item.

## Discussion

We conducted a scoping review of 70 prehabilitation RCTs, published from 1994 to 2022, to assess adherence to 6 checklists for reporting quality in the fields of exercise, nutrition, and psychosocial interventions. The overall mean agreement to these reporting guidelines was 57%. While adherence with CONSORT has improved over the last 3 decades, intervention reporting according to CERT, Modified-CERT, PRESENT 2020, and CONSORT-SPI remains at approximately 40–78% agreement without substantial improvement over time. This review is an important step to understand current practices and gaps in reporting that could be addressed to improve the quality of future reporting and transparency of published evidence for future randomized trials focused on prehabilitation.

The overall moderate agreement of 57% to existing reporting guidelines is meaningful because the lack of reporting of a specific item may represent that the item was not considered during the study planning and conduct. For instance, in accordance with the TIDieR guideline, we identified that compliance to the exercise intervention was only reported in 31 trials (45%). If intervention compliance was not reported, we postulate that it is unlikely that it was considered. Insufficient quality of reporting is neither new, nor unique to prehabilitation (Yamato et al. 2016; Hariohm et al. 2017; Hoffmann et al. 2013). An investigation into the completeness of reporting for RCTs of physical therapy interventions revealed that for intervention groups, 23% (*n* = 46) of trials did not describe half of the TIDieR items, and reporting was worse for control groups, as 75% (*n* = 149) of trials described less than half of the items listed in the guideline (Yamato et al. 2016).

Comparison of prehabilitation RCTs to the CONSORT 2010 checklist revealed that a clear hypothesis (item 2b) was reported in only 41% of prehabilitation RCTs. This is a surprising finding since the expected impact of an intervention is a main argument for justifying the trial to any granting agency, ethics committee, and the patients involved. This reporting is also a key component of a pre-registered protocol, which is crucial for a low risk-of-bias RCT. Furthermore, the accurate reporting of randomization methods, type, blinding and its implementation, and allocation concealment, was only reported in 65–80% of trials. Randomization and blinding represent another cornerstone to minimize bias in biomedical research. In research, bias occurs when systematic error is introduced into sampling (e.g., selection bias) or measurement (e.g., performance or detection bias) and leads to erroneous findings that deviate from the truth (Higgins et al. 2011). Concealed randomization reduces selection bias at trial entry and remains a crucial component of high-quality trials (Altman 1991). Likewise, intervention effects are consistently overestimated if the outcome assessor is not blinded (Saltaji et al. 2018). Since blinding of participants and people delivering the intervention is often impossible in RCTs of prehabilitation, strategies to mitigate the impact of unblinded assessments (e.g., a blinded assessor for the primary outcome alone) should be implemented to reduce bias and be reported with highest rigor possible to enhance trustworthiness of findings.

Prehabilitation trials reported 54% and 40% of the items in accordance with exercise interventions (CERT) and Modified-CERT, respectively. The information missing from prehabilitation trials included detailed descriptions of the interventions employed and how they were instructed (e.g., cues of modification and progression, specific sets, and repetitions) as well as if and how the interventions were tailored to patients. The discrepancy of 14% between CERT and Modified-CERT is likely because the Modified-CERT guideline, published by the *International Journal of Sports Physical Therapy* (IJSPT), requires even greater detailed description of exercise interventions. For example, while CERT item no. 9 states the following: “Content of any home program component” and was therefore reported in 93% of trials containing a home program, the Modified-CERT guideline requires specifics for the same item, “provide details on how the home program was instructed, delivered, and progressed throughout intervention.” This detailed description was reported by 27% of trials only. The poor descriptions of the prehabilitation exercise interventions were also reflected in the 44% agreement rate to the CONSORT 2010 item 5 “providing sufficient details to allow replication of interventions, including how and when they were actually administered.” This means, what part of the intervention was standardized and how much was adapted for individual patients, was not specified (e.g., progression of training intensity occurred when the participant could complete the aerobic exercise with mild exertion according to Borg 12) (Gillis et al. 2014). The ultimate goal of clinical research must be to translate findings into practice. Ambiguous descriptions of exercises do not inform the implementation of evidence-based interventions in real-world settings, and thus are not beneficial to the clinician nor the patient who cannot reproduce the intervention in clinic. Additionally, interventions cannot be further validated and generalized to a larger population if they cannot be replicated. As such, the IJSPT now requires all submissions to be accompanied by either the TIDieR checklist or the Modified-CERT checklist if exercise interventions are included in a manuscript (Page et al. 2017).

An important shortcoming of prehabilitation trials was the insufficient explanation on how the person delivering the intervention was trained, instructed, or had experience in the field/familiarity with a specific intervention that was then delivered to patients. Prehabilitation RCTs reported this applicable item (2) in only 15% and 8% of cases, according to CERT and Modified-CERT, respectively. Similarly, only 8% of trials with psychosocial interventions reported this applicable item (5a, CONSORT-SPI). According to these guidelines, a simple statement, such as kinesiologist, dietitian, or psychosocial personnel, does not sufficiently reflect a person’s expertise in a given field and should therefore be followed by a short declaration regarding years of training or experience. If there are multiple therapists, information should also be provided on how they were instructed and synchronized to assure homogeneity of delivering the intervention. Additionally, only 38% of all trials documented relevant harms and unintended consequences observed (PRESENT 15b). This is a limitation, since prehabilitation studies may entail rigorous exercise programs, such as high-intensity interval training (HIIT), for high-risk patients who may be at increased risk for adverse events.

When prescribing an intervention, the minimal threshold for successful completion of a program (e.g., attendance of 75% to all prescribed training sessions) or therapeutic target (e.g., 1.2-g protein/kg) (Weimann et al. 2021) required to reach a positive effect should be pre-defined (Church et al. 2007). Defining this threshold or target a priori permits evaluation of whether an intervention was completed successfully or not. For example, if an intervention consists of “walking at moderate intensity for 30 minutes 5 days per week,” at what point is the exercise completed successfully? Does walking 5 times per week at a low intensity count as successful completion of the intervention? How was intensity measured and monitored? All of the above markers of success must be defined before the initiation of a trial (Modified-CERT item 16) and should be followed by diligent assessment of adherence (how, when, and by whom). We identified that only 18% of prehabilitation trials reported markers of success, and 35 (CERT, 16)–45% (TIDieR, 12) of trials with an exercise intervention reported intervention adherence. Summarizing and reporting data on the effectiveness of an intervention alone, without consideration of implementation factors (e.g., prescription adherence, training, or experience of study team) limits the conclusions that can be drawn. Success or failure of an intervention could be the result of its efficacy, its implementation, or a combination of the two (Proctor et al. 2011). If the implementation factors are not reported, it is difficult to discern where success or failure lies. This makes future (successful) uptake of the intervention in clinical practice a challenge.

Surprisingly, we identified that the prehabilitation literature has underreported preoperative patient characteristics known for having a negative impact on perioperative outcomes and for producing variation in response to treatment (Gillis et al. 2021). Screening for malnutrition, frailty, and sarcopenia were reported in a minority of cases: 11%, 6%, and 0%, respectively. Yet, malnutrition has been found to modify response to prehabilitation. By failing to give an intervention to those who need it, or to stratify findings by patient characteristics, the prehabilitation effect could be diluted, and negative outcomes can be (wrongfully) reported leading to abandonment of the intervention (Gillis et al. 2022).

Given the complex and multidisciplinary nature of prehabilitation, we believe the development of a prehabilitation-specific reporting guideline is a relevant next step to improve the quality of evidence in this field. A reporting guideline for prehabilitation would allow researchers to plan and report trials in accordance with the critical aspects of intervention reporting, including multimodal components (e.g., nutrition, exercise, psychosocial, smoking cessation, anemia correction), the timing of the intervention, the duration, and the outcomes measured (including stratification by patient subgroups). Such a checklist may provide researchers and healthcare providers with clear, standardized criteria for reporting prehabilitation interventions and outcomes, increasing the quality and completeness of reporting, which ultimately could improve the quality of evidence regarding the value of prehabilitation in perioperative care.

### Strengths and limitations

To our knowledge, this is the first review to investigate the current standards of RCT reporting of prehabilitation and address a clear lack of reporting consistency in the literature. Our review is limited to trials published in English or French and thus may be subject to language bias. In addition, we only included trials using interventions labelled as “prehabilitation” that met our definition and may have omitted relevant trials where this term was not used. Another limitation is that some of the explanations for the items of the guidelines were ambiguous, making it difficult to determine if an item was met or not, especially if an item contained multiple points (e.g., TIDieR item 11: if intervention agreement or fidelity was assessed, describe how and by whom, and if any strategies were used to maintain or improve fidelity). Additionally, for some items (e.g., important changes to methods (CONSORT 2010 3b) after trial commencement), it was difficult to discern whether an item was not reported because it was not applicable, or it was simply omitted. These limitations were mitigated by having 2 independent reviewers conduct data extraction, and through discussion with the study team when needed, to attain consistency. The a priori subgroup analyses performed to evaluate the evolution in reporting quality over time (Supplemental material 3) were divided by decades, which lead to an uneven distribution of the included studies, and does not consider the year the respective checklists were published (CONSORT 2010, TIDieR 2014, CERT 2016, Mod-CERT 2017, CONSORT-SPI 2018, PRESENT 2020). Finally, we were unable to find a guideline for the reporting of nutrition interventions in RCTs. Even PRESENT 2020, a guideline for reporting of evidence in sport and exercise nutrition trials, does not specifically cover relevant elements such as nutritional assessment, intervention description, or outcome assessment.

## Conclusion

In accordance with available reporting guidelines, mean, overall reporting of research methods and intervention details in prehabilitation trials is suboptimal. While the reporting of trial methods appears to be improving with time, no such improvement has been observed in reporting of prehabilitation interventions. That said, prehabilitation interventions, especially when multimodal and personalized to meet individual patient needs, are complex in nature, and a single reporting guideline that meets this complexity does not currently exist. In biomedical research, there are several guidelines for appropriate reporting in various fields that could be adapted for prehabilitation. We suggest that, in the future, reporting might improve with the development of a reporting checklist focused on prehabilitation methods, intervention components, and outcomes.

### Supplementary Information

**Additional file 1: Supplementary Material 1.** List of checklist items for CONSORT 2010, TIDieR, CERT, Modified-CERT, PRESENT 2020, CONSORT-SPI 2018.

**Additional file 2: Supplementary Material 2.** Literature Search.

**Additional file 3: Supplementary Material 3.** Figure of agreement ratio over last decades.

## Data Availability

The data that support the findings of this study are available from the corresponding author, C. G., upon reasonable request.
