# Supplementary material for: Reporting quality of randomized controlled trials in prehabilitation: a scoping review
Source: Perioper Med (Lond). 2023 Aug 31;12:48. doi: 10.1186/s13741-023-00338-8 (PMC10472732; doi:10.1186/s13741-023-00338-8)
Supplement: Supplementary file 1 — Additional file 1: Supplementary Material 1. List of checklist items for CONSORT 2010, TIDieR, CERT, Modified-CERT, PRESENT 2020, CONSORT-SPI 2018. [file 13741_2023_338_MOESM1_ESM.docx]

**Supplementary Material 1**: List of checklist items for CONSORT 2010, TIDieR, CERT, Modified-CERT, PRESENT 2020, CONSORT-SPI 2018

Consolidated Standards of Reporting of Trials (CONSORT 2010)

| 1a | Identification as a randomised trial in the title |
| --- | --- |
| 1b | Structured summary of trial design, methods, results and conclusions. |
| 2a | Scientific background and explanation of rationale |
| 2b | Specific objectives |
| 2b | or hypotheses |
| 3a | Description of trial design (such as parallel, factorial) including allocation ratio |
| 3b | Important changes to methods after trial commencement (such as eligibility criteria), with reasons |
| 4a | Eligibility criteria for participants |
| 4b | Settings and locations where the data were collected |
| 5 | The interventions for each group with sufficient details to allow replication, including how and when they were actually administered |
| 6 | Any changes to trial outcomes after the trial commenced, with reasons |
| 7a | How sample size was determined |
| 7b | When applicable, explanation of any interim analyses and stopping guidelines |
| 8a | Method used to generate the random allocation sequence |
| 8b | Type of randomisation; details of any restriction (such as blocking and block size) |
| 9 | Mechanism used to implement the random allocation sequence (such as sequentially numbered containers), describing any steps taken to conceal the sequence until interventions were assigned |
| 10 | Mechanism used to implement the random allocation sequence (such as sequentially numbered containers), describing any steps taken to conceal the sequence until interventions were assigned |
| 11a | If done, who was blinded after assignment to interventions (for example, participants, care providers, those assessing outcomes) and how |
| 11b | If relevant, description of the similarity of interventions |
| 12a | Statistical methods used to compare groups for primary and secondary outcomes |
| 12b | Methods for additional analyses, such as subgroup analyses and adjusted analyses |
| 13a | For each group, the numbers of participants who were randomly assigned, received intended treatment, and were analysed for the primary outcome |
| 13b | For each group, losses and exclusions after randomisation, together with reasons |
| 14a | Dates defining the periods of recruitment and follow-up |
| 14b | Why the trial ended or was stopped |
| 15 | A table showing baseline demographic and clinical characteristics for each group |
| 16 | For each group, number of participants (denominator) included in each analysis and whether the analysis was by original assigned groups |
| 17a | For each primary and secondary outcome, results for each group, and the estimated effect size and its  precision (such as 95% confidence interval) |
| 17b | For binary outcomes, presentation of both absolute and relative effect sizes is recommended |
| 18 | Results of any other analyses performed, including subgroup analyses and adjusted analyses, distinguishing  pre-specified from exploratory |
| 19 | All important harms or unintended effects in each group |
| 20 | Trial limitations, addressing sources of potential bias, imprecision, and, if relevant, multiplicity of analyses |
| 21 | Generalisability (external validity, applicability) of the trial findings |
| 22 | Interpretation consistent with results, balancing benefits and harms, and considering other relevant evidence |
| 23 | Registration number and name of trial registry |
| 24 | Where the full trial protocol can be accessed, if available |
| 25 | Sources of funding and other support (such as supply of drugs), role of funders |

Template for intervention description and replication (TIDieR 2014)

| 1 | Provide the name or a phrase that describes the intervention. (e.g prehabilitation) |
| --- | --- |
| 2 | Describe any rationale, theory, or goal of the elements essential to the intervention |
| 3a | Materials: Describe any physical or informational materials used in the intervention, including those provided to participants or used in intervention delivery or in training of intervention providers |
| 3b | Provide information on where the materials can be accessed (e.g. online appendix, URL) |
| 4 | Procedures: Describe each of the procedures, activities, and/or processes used in the intervention, including any enabling or support activities |
| 5 | For each category of intervention provider (e.g. psychologist, nursing assistant), describe their expertise, background and any specific training given |
| 6 | Describe the modes of delivery (e.g. face-to-face or by some other mechanism, such as internet or telephone) of the intervention and whether it was provided individually or in a group |
| 7 | Describe the type(s) of location(s) where the intervention occurred, including any necessary infrastructure or relevant features |
| 8 | Describe the number of times the intervention was delivered and over what period of time including the number of sessions, their schedule, and their duration, intensity or dose |
| 9 | If the intervention was planned to be personalised, titrated or adapted, then describe what, why, when, and how |
| 10 | If the intervention was modified during the course of the study, describe the changes (what, why, when, and how) |
| 11 | Planned: If intervention adherence or fidelity was assessed, describe how and by whom, and if any strategies were used to maintain or improve fidelity, describe them |
| 12 | Actual: If intervention adherence or fidelity was assessed, describe the extent to which the intervention was delivered as planned |

Consensus on exercise reporting template (CERT 2016)

| 1 | Type of exercise equipment |
| --- | --- |
| 2 | Qualifications, teaching/supervising experience, and/or training of the exercise instructor |
| 3 | Whether exercises are performed individually or in a group |
| 4 | Whether exercises are supervised or unsupervised |
| 5 | Measurement and reporting of adherence to exercise |
| 6 | Details of motivation strategies (e.g. phone calls) |
| 7 | Decision rules for progressing the exercise program |
| 8 | Each exercise is described so that it can be replicated (e.g., illustrations, photographs) |
| 9 | Content of any home program component |
| 10 | Non-exercise components (e.g. participants in the experimental group also received health coaching via telephone.) |
| 11 | How adverse events that occur during exercise are documented and managed |
| 12 | Setting in which exercises are performed |
| 13 | Detailed description of the exercises (e.g. sets, repetitions, duration, intensity) |
| 14 | Whether exercises are generic (“one size fits all”) or tailored to the individual |
| 15 | Decision rules that determines the starting level for exercise Provide how specific sets, repetitions, resistances are determined initially, including the home program |
| 16 | Whether the exercise intervention is delivered and performed as planned |

Modified Consensus on exercise reporting template (Modified CERT 2017)

| 1 | Provide equipment manufacturer, city, state, country, if appropriate, and appropriate copyright |
| --- | --- |
| 2 | If exercise program is administered by multiple therapists, provide detail on how each therapist was trained in the intervention |
| 3 | If group exercise, note the size of the group |
| 4 | Note if exercise is ‘direct’ one-onone or indirect supervision |
| 5 | Provide exercise log in appendix, or specify method for both inclinic and home program compliance recording |
| 6 | Note behavioral strategies to improve compliance with home exercise program (See #10) |
| 7 | Provide criteria for progression of each exercise both in the clinic and in home program (See #13) |
| 8 | Provide detailed instructions (including cues and modifications) for each exercise, including patient booklets in a table, appendix, or supplement. Avoid using only exercise names as descriptors. |
| 9 | Provide details on how home program was instructed (i), delivered (d), and progressed (p) throughout intervention (Note # 1, 5, 7, 8, 11, 13, 14, 15) |
| 10 | Include education (posture, ergonomics, modalities) in appendix or where materials can be accessed |
| 11 | Reported and addressed in limitations or future research considerations |
| 12 | Note which exercises were performed in clinic and/or home |
| 13 | Compliments #7, and provide progression rules for individual exercises, including the home exercise program. Do not simply refer to the protocol based on the name of the developer. |
| 14 | If tailored, detail how decisions are made for choosing/progressing exercises, including options for therapist. Provide algorithm or flow chart for tailored exercises. |
| 15 | Provide how specific sets, repetitions, resistances are determined initially, including the home program (not applicable if aerobic only) |
| 16 | Define markers of “success” (compliance, outcomes) |

Proper Reporting of Evidence in Sport and Exercise Nutrition Trials (PRESENT 2020)

| 1a | State the independent (groups/conditions) and dependent (outcome) variables |
| --- | --- |
| 1b | Identify the study population or case |
| 2a | Specify the research design, methods, and characteristics of study population |
| 2b | Report a balanced account of the results and cite actual data |
| 2c | Restrict conclusions to measured variables, without speculation or unsupported recommendations |
| 3a | Present a scientific rationale based on an objective review of available evidence |
| 3b | State the aims, objectives, research questions, and/or hypotheses |
| 4 | Provide details of ethical approval (citing conduct of human research in accordance with the Declaration of Helsinki) |
| 5 | Summarize the research design (e.g., parallel trial/cross-over, randomized, counterbalanced, blinding, observational) |
| 6a | List the eligibility (inclusion/exclusion) criteria and sampling method |
| 6b | Characterize the study sample (e.g., demographics, anthropometry, lifestyle) |
| 6c | Report the setting/location and periods of recruitment and data collection |
| 6d | Justify the sample size (presenting the selected target effect size and error variances to replicate sample size estimates) |
| 7 | Detail all aspects of the groups/conditions (considering the need to verify the composition of ingested substances) |
| 8a | Define the pre-specified primary, secondary and/or mechanistic outcome variables |
| 8b | Rationalize the selection of test protocols, considering validity and reliability (e.g., coefficient of variation, familiarization) |
| 8c | Justify the smallest worthwhile effect or minimal clinically important difference (MCID) |
| 9 | Detail the exact mechanisms of generating and concealing the random allocation sequence |
| 10 | Document whether participants and/or researchers were aware of allocation (e.g., exit questionnaire) |
| 11 | Describe within- and between-participant controls (e.g., replication/reporting of diet, physical activity, sleep, menstrual cycle) |
| 12 | Detail control of systematic influences of serial measurements (e.g., sequence effect in analysis model, wash-out interval) |
| 13a | Specify the contrast for primary inferences (i.e., relative to the appropriate control, not changes from baseline in each group/condition) |
| 13b | Clearly distinguish and fully justify any unplanned, interim or exploratory subgroup analyses |
| 13c | Describe any adjustments for violated statistical assumptions and for relevant covariates (e.g., baseline measures) |
| 14a | Report the sample size at each phase from recruitment to analysis (with reasons for losses and exclusions) |
| 14b | Ensure data analysis matches research design, avoiding data pooling across groups/conditions (i.e., pseudoreplication) |
| 15 | Report SI units and report measures of central tendency, variability, and effect size/precision (confidence intervals) |
| 15b | Report individual data/responses (e.g., draw figures showing the raw data in each group/condition) |
| 15c | Document all relevant harms and unintended consequences observed |
| 16a | Present an objective and balanced interpretation of the observed data within the context of existing evidence |
| 16b | Consider the applicability and/or practical relevance of the research findings (e.g., external validity) |
| 16c | Acknowledge strengths and limitations of the research relevant to accurate interpretation (e.g., internal validity) |
| 17 | State any relevant relationships (e.g., financial, technical, material support) |
| 18 | Identify any publicly registered or published protocol (explaining any deviations) |

CONSORT extension for psychosocial interventions (CONSORT SPI 2018)

| 1b | Refer to CONSORT extension for social and psychological intervention trial abstracts |
| --- | --- |
| 2b | If pre-specified, how the intervention was hypothesized to work |
| 4a | When applicable, eligibility criteria for settings and those delivering the interventions |
| 5a | Extent to which interventions were actually delivered by providers and taken up by participants as planned |
| 5b | Where other informational materials about delivering the intervention can be accessed |
| 5c | When applicable, how intervention providers were assigned to each group |
| 12a | How missing data were handled, with details of any imputation method |
| 13a | Where possible, the number approached, screened, and eligible prior to random assignment, with reasons for non-enrolment |
| 15 | Include socioeconomic variables where applicable |
| 17a | Indicate availability of trial data |
| 25 | Declaration of any other potential interests |
| 26a | Any involvement of the intervention developer in the design, conduct, analysis, or reporting of the trial |
| 26b | Other stakeholder involvement in trial design, conduct, or analyses |
| 26c | Incentives offered as part of the trial |
