# Supplementary material for: Reporting quality of randomized controlled trials in prehabilitation: a scoping review
Source: Perioper Med (Lond). 2023 Aug 31;12:48. doi: 10.1186/s13741-023-00338-8 (PMC10472732; doi:10.1186/s13741-023-00338-8)
Supplement: Supplementary file 2 — Additional file 2: Supplementary Material 2. Literature Search. [file 13741_2023_338_MOESM2_ESM.docx]

**Supplementary Material 2: Literature Search**

Document updated: Date: March 25, 2022 by Genevieve Gore

Database searches conducted: Date: March 22, 2022 by Genevieve Gore

Database searches peer reviewed: NA

Database searches updated: Date: by

Grey Literature searches conducted NA by

| Platform | Database(s) | Database coverage dates | # Results | Search Date | Saved (account) | Remarks |
| --- | --- | --- | --- | --- | --- | --- |
| Ovid | Ovid MEDLINE ALL(R) | 1946 - | 384 | 2022/03/25 | gengore | *Reviews, editorials, historical articles, and records with case report in the title excluded*  *Studies indexed as animal-only excluded*  *Studies indexed as child-only excluded*  *Limited to English or French* |
| Ovid | EMBASE Classic + EMBASE | 1947 - | 383 | 2022/03/25 | gengore | *Excluded articles with case report/meta analysis/scoping review/systematic review) in title and the following publication types: Conference abstract/conference proceeding/"conference review"/editorial/review*  *Limited to English or French* |
| Ovid | APA PsycInfo | 1806 - | 37 | 2022/03/25 | gengore | *No exclusions given small set of results* |
| Web of Science | SCI-EXP, SSCI, ESCI | 1900 - | 420 |  |  | *Reviews, conference abstracts, and editorials excluded* |
| EBSCOhost | CINAHL | 1937 - | 161 | 2022/03/25 | NA | *Case Study, Editorial, Historical Material, Meta Analysis, Meta Synthesis, Review, Systematic Review publication types, and records with case report, meta analysis, scoping review or systematic review in the title excluded*  *Studies indexed as animal-only excluded*  *Limited to English or French* |
| Cochrane Library | CENTRAL (Trials) | Inception - | 558 | 2022/03/22 | NA | *No limits used* |
| JBI |  |  |  |  |  | Database omitted: Includes summarized and appraised evidence |
| Total |  |  | 1943 total |  |  |  |
|  |  |  |  |  |  |  |

**Limits or filters used:**

**MEDLINE search includes a combination of the Cochrane sensitive search for RTCs combined with the SIGN search for RCTs**

**Citation for search filter used for CINAHL:**

Glanville J, Dooley G, Wisniewski S, Foxlee R, Noel‐Storr A. Development of a search filter to identify reports of controlled clinical trials within CINAHL Plus. Health Information & Libraries Journal. 2019 Mar;36(1):73-90.

**Original searches: [copy and paste the search strategies here]**

Ovid MEDLINE(R) ALL <1946 to March 24, 2022>

Search run on March 25, 2022

1 (prehab* or pre-hab* or prerehab* or pre-rehab*).tw,kf. 1319

2 ((preoperative* or pre-operative*) adj rehab*).tw,kf. 151

3 or/1-2 1446

4 Randomized Controlled Trials as Topic/ 153780

5 randomized controlled trial/ 562420

6 Random Allocation/ 106788

7 Double Blind Method/ 170833

8 Single Blind Method/ 31741

9 clinical trial/ 534473

10 clinical trial, phase i.pt. 23481

11 clinical trial, phase ii.pt. 37481

12 clinical trial, phase iii.pt. 20254

13 clinical trial, phase iv.pt. 2301

14 controlled clinical trial.pt. 94763

15 randomized controlled trial.pt. 562420

16 multicenter study.pt. 318062

17 clinical trial.pt. 534473

18 exp Clinical Trials as topic/ 371969

19 (clinical adj trial$).tw,kf. 440698

20 ((singl$ or doubl$ or treb$ or tripl$) adj (blind$3 or mask$3)).tw,kf. 187222

21 placebos/ 35911

22 placebo$.tw,kf. 234881

23 randomi?ed.tw,kf. 719184

24 randomly.tw,kf. 379433

25 (trial or groups).ab. 2761763

26 or/4-25 4083357

27 case report.ti. 275934

28 editorial/ 599217

29 historical article/ 368034

30 systematic review/ or (scoping review or systematic review).ti. 241470

31 review.pt. 2956462

32 meta analysis/ or meta analysis.ti. 192409

33 or/27-32 4285923

34 26 not 33 3604756

35 34 not (exp animals/ not humans.sh.) 3094403

36 35 not ((exp infant/ or exp child/ or adolescent/) not exp adult/) 2836810

37 3 and 36 390

38 limit 37 to (english or french) 384

Embase Classic+Embase <1947 to 2022 March 24>

Search run on March 25, 2022

1 (prehab* or pre-hab* or prerehab* or pre-rehab*).ti,ab,kf. 2073

2 ((preoperative* or pre-operative*) adj rehab*).ti,ab,kf. 240

3 1 or 2 2280

4 "randomized controlled trial (topic)"/ 222905

5 Randomized Controlled Trial/ 703638

6 Randomization/ 93586

7 Double Blind Procedure/ 195993

8 single blind procedure/ 45621

9 placebo/ 389016

10 (random allocation or multicenter study or multicentre study or (clinical adj trial*) or ((singl* or doubl* or treb* or tripl*) adj (blind* or mask*)) or placebo* or randomi?ed or randomly).tw,kf. or (trial or groups).ab. 4990871

11 or/4-10 5281691

12 3 and 11 852

13 12 not (exp meta analysis/ or exp review/ or (case report or meta analysis or scoping review or systematic review).ti. or (conference abstract or conference proceeding or "conference review" or editorial or review).pt.) 398

14 limit 13 to (english or french) 383

APA PsycInfo <1806 to March Week 3 2022>

Search run on March 25, 2022

1 (prehab* or pre-hab* or prerehab* or pre-rehab*).ti,ab. 88

2 ((preoperative* or pre-operative*) adj rehab*).ti,ab. 5

3 1 or 2 92

4 exp randomized controlled trials/ 1156

5 clinical trials/ 12039

6 placebo/ 6205

7 treatment effectiveness evaluation/ 26555

8 exp treatment outcomes/ 131363

9 followup studies/ 12390

10 (random allocation or multicenter study or multicentre study or (clinical adj trial*) or ((singl* or doubl* or treb* or tripl*) adj (blind* or mask*)) or placebo* or randomi?ed or randomly).tw. or (trial or groups).ab. 731246

11 or/4-10 850773

12 3 and 11 37

Science Citation Expanded (SCI-EXP), Social Sciences Citation Index (SSCI), Emerging Sources Citation Index (ESCI)

Search run on March 25, 2022

420 results

(TI=(("prehab*" or "pre-hab*" or "prerehab*" or "pre-rehab*") OR (("preoperative*" or "pre-operative*") NEAR/0 "rehab*")) OR AB=(("prehab*" or "pre-hab*" or "prerehab*" or "pre-rehab*") OR (("preoperative*" or "pre-operative*") NEAR/0 "rehab*")) OR AK=(("prehab*" or "pre-hab*" or "prerehab*" or "pre-rehab*") OR (("preoperative*" or "pre-operative*") NEAR/0 "rehab*"))) AND (TS=("random allocation" OR "multicenter study" OR "multicentre study" OR (clinical NEAR/0 trial*) OR ((singl* or doubl* or treb* or tripl*) NEAR/0 (blind* or mask*)) OR placebo* OR randomi$ed OR randomly) OR AB=("trial" or "groups")) and Review Articles or Editorial Materials or Meeting Abstracts (Exclude – Document Types)

Cochrane Library

Search run on March 25, 2022 (CDT)

Search Name:

Date Run: 26/03/2022 02:39:01

Comment:

ID Search Hits

#1 (prehab* or pre next hab* or prerehab* or pre next rehab* or ((preoperative* or pre-operative*) next rehab*)):ti,ab,kw in Trials 558

CINAHL (EBSCOhost)

Search run on March 25, 2022 (CDT)

| Friday, March 25, 2022 8:45:30 PM |
| --- |

| **#** | **Query** | **Limiters/Expanders** | **Last Run Via** | **Results** |
| --- | --- | --- | --- | --- |
| S10 | S8 AND S9 | Expanders - Apply equivalent subjects Search modes - Boolean/Phrase | Interface - EBSCOhost Research Databases Search Screen - Advanced Search Database - CINAHL Plus with Full Text | 161 |
| S9 | LA English OR French | Expanders - Apply equivalent subjects Search modes - Boolean/Phrase | Interface - EBSCOhost Research Databases Search Screen - Advanced Search Database - CINAHL Plus with Full Text | 8,026,079 |
| S8 | S7 NOT (S5 OR S6) | Expanders - Apply equivalent subjects Search modes - Boolean/Phrase | Interface - EBSCOhost Research Databases Search Screen - Advanced Search Database - CINAHL Plus with Full Text | 162 |
| S7 | S3 AND S4 | Expanders - Apply equivalent subjects Search modes - Boolean/Phrase | Interface - EBSCOhost Research Databases Search Screen - Advanced Search Database - CINAHL Plus with Full Text | 199 |
| S6 | TI case report OR meta analysis OR scoping review OR systematic review | Limiters - Publication Type: Case Study, Editorial, Historical Material, Meta Analysis, Meta Synthesis, Review, Systematic Review Expanders - Apply equivalent subjects Search modes - Boolean/Phrase | Interface - EBSCOhost Research Databases Search Screen - Advanced Search Database - CINAHL Plus with Full Text | 135,654 |
| S5 |  | Limiters - Publication Type: Case Study, Editorial, Historical Material, Meta Analysis, Meta Synthesis, Review, Systematic Review Expanders - Apply equivalent subjects Search modes - Boolean/Phrase | Interface - EBSCOhost Research Databases Search Screen - Advanced Search Database - CINAHL Plus with Full Text | 1,276,321 |
| S4 | (randomized controlled trials OR MH double-blind studies OR MH single-blind studies OR MH random assignment OR MH pretest-posttest design OR MH cluster sample OR TI (randomised OR randomized) OR AB (random*) OR TI (trial) OR (MH (sample size) AND AB (assigned OR allocated OR control)) OR MH (placebos) OR PT (randomized controlled trial) OR AB (control W5 group) OR MH (crossover design) OR MH (comparative studies) OR AB (cluster W3 RCT)) NOT ((MH animals+ OR MH animal studies OR TI animal model*) NOT MH human) | Expanders - Apply equivalent subjects Search modes - Boolean/Phrase | Interface - EBSCOhost Research Databases Search Screen - Advanced Search Database - CINAHL Plus with Full Text | 904,609 |
| S3 | S1 OR S2 | Expanders - Apply equivalent subjects Search modes - Boolean/Phrase | Interface - EBSCOhost Research Databases Search Screen - Advanced Search Database - CINAHL Plus with Full Text | 792 |
| S2 | ((preoperative* OR pre-operative*) W1 rehab*) | Expanders - Apply equivalent subjects Search modes - Boolean/Phrase | Interface - EBSCOhost Research Databases Search Screen - Advanced Search Database - CINAHL Plus with Full Text | 116 |
| S1 | (prehab* OR pre-hab* OR prerehab* OR pre-rehab*) | Expanders - Apply equivalent subjects Search modes - Boolean/Phrase | Interface - EBSCOhost Research Databases Search Screen - Advanced Search Database - CINAHL Plus with Full Text | 699 |
