# Supplementary material for: Reporting quality of randomized controlled trials in prehabilitation: a scoping review
Source: Perioper Med (Lond). 2023 Aug 31;12:48. doi: 10.1186/s13741-023-00338-8 (PMC10472732; doi:10.1186/s13741-023-00338-8)
Supplement: Supplementary file 3 — Additional file 3: Supplementary Material 3. Figure of agreement ratio over last decades. [file 13741_2023_338_MOESM3_ESM.docx]

**Supplementary Material 3:** Figure of agreement ratio over last decades


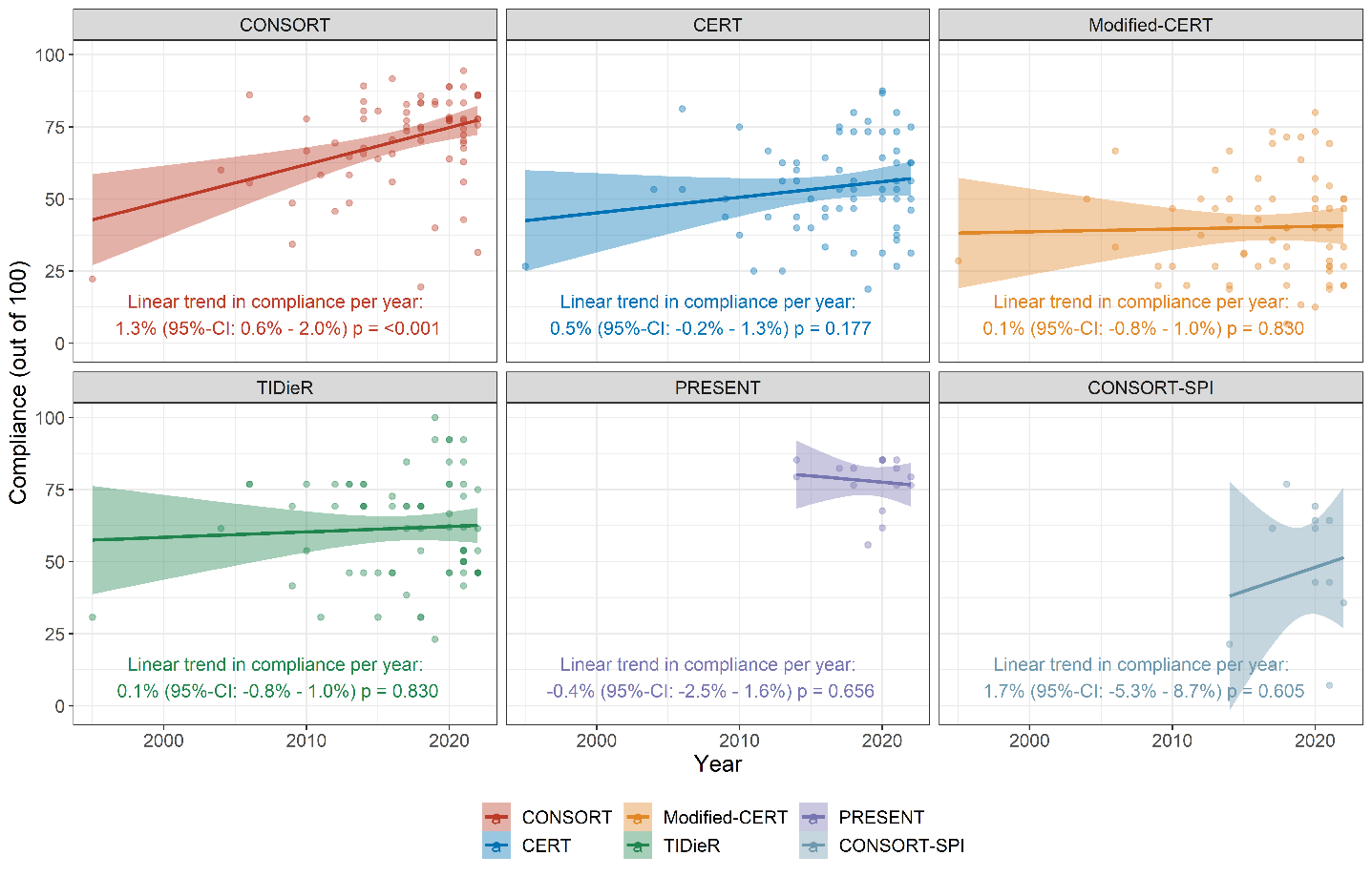


Table of agreement over last decade: mean and standard deviation of agreement ratio in % over the last decades.

|  | **Decade 1993-2003** | **Decade 2004-2013** | **Decade 2014-2022** |
| --- | --- | --- | --- |
| **CONSORT 2010** | 22.2 (-) [N=1] | 59.5 (13.9) [N=13] | 74.1 (14.4) [N=56] |
| **CERT 2016** | 26.7 (-) [N=1] | 51.8 (17.2) [N=13] | 55.3 (16.2) [N=51] |
| **Modified-CERT 2017** | 28.6 (-) [N=1] | 39.3 (15.2) [N=13] | 40.6 (18.7) [N=51] |
| **TiDER 2014** | 30.8 (.) [N=1] | 64.2 (16.0) [N=13] | 61.6 (17.9) [N=56] |
| **PRESENT 2020** | - | - | 77.9 (8.92 [N=16] |
| **CONSORT-SPI 2018** | - | - | 46.8 (23.1) [N=12] |

Consolidated standards of reporting of trials, CONSORT 2010; TIDieR, Template for intervention description and replication 2014, Consensus on exercise reporting template, CERT 2016; Modified-CERT 2017; Proper reporting of evidence in sport and exercise nutrition trials, PRESENT 2020; CONSORT extension for psychosocial interventions, CONSRT-SPI 2018
